# Psychometric validation of the Chinese version of the Adolescent Asthma Self-Efficacy Questionnaire

**DOI:** 10.3389/fpsyg.2022.1013989

**Published:** 2022-12-22

**Authors:** Yuanyuan Wang, Hongyu Chen, Jinjin Cao, Mei Li, Jianan Wang, Ruochen Jing

**Affiliations:** ^1^Department of Nursing, Children's Hospital of Nanjing Medical University, Nanjing, China; ^2^Department of Neurosurgery, The First Hospital of China Medical University, Shenyang, China; ^3^Department of Gastroenterology, Children's Hospital of Nanjing Medical University, Nanjing, China

**Keywords:** self-efficacy, asthma, adolescent, reliability, validity, Chinese

## Abstract

**Background:**

Self-efficacy was considered as a promising target for the self-management of symptoms for adolescents with asthma. The measurement of self-efficacy in adolescents with asthma requires effective self-report tools, which have not been met with at present. So, the aim of this study was to cross-culturally validate the Adolescent Asthma Self-Efficacy Questionnaire (AASEQ).

**Methods:**

As many as 408 adolescents with asthma were invited to take up the psychometric properties test between July 2021 and June 2022. We conducted the confirmatory factor analysis (CFA) to determine the structure of the AASEQ. The relationship between the AASEQ and General Self-Efficacy Scale was tested to evaluate the construct validity. The reliability was evaluated by retest reliability, internal consistency, and interfactor correlation.

**Results:**

The results of the present study showed that the confirmatory factor analysis indicated a significantly good fit for a four-factor model, which explained 62.697% of the total variance. The fit indices of the four-factor model were acceptable, and the standardized factor loading ranged from 0.631 to 0.880. The C-AASEQ showed an acceptable internal consistency (Cronbach's α = 0.810–0.927) and test-retest reliability (intraclass correlation coefficient = 0.64–0.89). Content validity index at the scale level was 0.96, and content validity index at the item level was 0.86 to 1.

**Conclusion:**

The Chinese version of Adolescent Asthma Self-Efficacy Questionnaire showed consistently acceptable positive psychometric properties and can be used as an instrument to assess the self-efficacy of adolescents with asthma in China, as corroborated in the present study.

## Introduction

Asthma is the most common chronic respiratory disease affecting more than 300 million people worldwide (Stern et al., 2020). It is not a single disease, but a complex syndrome, which is one of the most common long-term diseases that have a significant impact on adolescents (Holley et al., 2017). The asthma prevalence, mortality, and severity vary all over the world with a prevalence that ranges from 4.1 to 32% (Stern et al., 2020), and the proportion of asthma in adolescents increases by 0.28% annually (Asher et al., 2020). Although many patients have achieved good control over the disease through current therapies, prognosis of asthma after treatment is not as good as expected. It is estimated that 5%−10% of patients still suffer from asthma relevant symptoms despite receiving several treatments to get cured of the disease (Fleming et al., 2015). Asthma has brought about many adverse effects on young patients, such as a poor quality of life (QoL) (Cekic et al., 2021) and an impaired lung function (McGeachie et al., 2016). They are also vulnerable to the side effects of drugs (Kaur and Singh, 2018; Haktanir Abul and Phipatanakul, 2019). In addition, adolescent asthma brings a huge burden to national medical care (Perry et al., 2019).

As far as we know, the pathophysiology and risk factors of asthma are multifactorial and are closely related to genetic, environmental, psychological, and socioeconomic conditions (Jones et al., 2022). If the adolescents come from socially vulnerable groups, such as from a poor economically downtrodden family and live under conditions with less or no access to education, which may affect the use of medical facilities and medicinal drugs, their exposure to allergens will increase, resulting in an increase in the risk of asthma attacks (Kopel et al., 2014). Research shows that imparting asthma-related education to adolescents can effectively bring down the frequency of making several visits to hospital (Walders et al., 2006), apart from providing adolescents with requisite knowledge and offering them adequate technical support to improve their disease awareness and self-management ability to tackle the disease. It is worth noting that there is a strong two-way relationship between the mental health of patients with chronic diseases and disease control (Fernandes et al., 2010; Griffiths et al., 2021). At present, research has identified several psychological and behavioral factors that affect adolescents' asthma self-management, such as forgetting to take treatment, lack of adequate knowledge of asthma, heavy treatment burden, false beliefs, embarrassment of suffering from asthma, and communication difficulties with healthcare providers (Holley et al., 2017). In the pathogenesis of asthma, emotional factors, such as stress, anxiety, and other negative emotions, may negatively affect the evolution of asthma, trigger an asthma attack, or exacerbate asthma symptoms (Sastre et al., 2018). When asthma disease gets aggravated, the related asthma symptoms also deteriorate rapidly, and severe asthma symptoms are often chronic and even frightening to the adolescents, leading to health-related anxiety and high vigilance, which may be the future inducement of asthma (Kew et al., 2016). Asthmatic depression will lead to poor drug compliance and non-compliance with the recommended lifestyle, which may increase the probability of asthma attacks and the possibility of losing control over asthma (Kulikova et al., 2021). Therefore, studies have shown that promoting the positive belief of an asthmatic adolescent to enhance their understanding of and confidence in disease management serves as an effective non-drug therapy for the treatment of asthma in adolescents (Pateraki and Morris, 2018).

Self-efficacy, which has been defined as “an individual's belief that he is feeling capable of organizing their work and believe they can be successful in accomplishing certain tasks” according to Bandura (Bandura, 1977), is thought to be a promising target for asthma therapy. For many diseases, self-efficacy is an important part of disease self-management and also a major influencing factor to implement the required behavior (White et al., 2017). Results from previous studies have shown that self-efficacy is significantly correlated with outcomes of disease treatment, such as cancer (Kwak et al., 2021), functional constipation (Santucci et al., 2020), inflammatory bowel diseases (Carlsen et al., 2017), type 1 diabetes (Bassi et al., 2020), and cystic fibrosis (Faint et al., 2017). In the case of asthma, studies have shown similar results that self-efficacy is associated with better control of asthma symptoms and has helped to improve the quality of life (Lavoie et al., 2008; Sleath et al., 2022). It also covers the preparation for change and motivation for drug persistence, which are considered to be effective strategies for symptom management (Barikani et al., 2021).

Due to the key role played by self-efficacy in asthma self-management, it is essential to measure this concept fully for formulating intervention measures and measuring the effectiveness of these intervention measures. The tools that were involved in the evaluation of adolescent asthma self-efficacy in previous studies were general measuring tools and subscales of a full scale, such as general self-efficacy scale (GSES) (Schwarzer and Jerusalem, 1995), asthma outcome expectation (AOE-SE), asthma self-efficacy scale (ASE), and asthma management index (AMI-SE) (Bursch et al., 1999; Rhee et al., 2020). However, general measuring tools are not comprehensive enough to measure the self-efficacy of specific diseases, much less asthma. For the other three specific scales, the scope of AOE-SE is limited to specific clinical tasks, including compliance with treatment and clinical appointments, and identification of symptoms/triggers. However, exercise restriction and emotion are also important deciding components on the quality of life of asthmatic adolescents, and the AOE-SE lacks the predictive ability in this regard. In many AMI-SE projects, the focus alwayd seems to be on bestowing maximum attention to various actions of asthma management, rather than on the “sense of confidence” in implementing these actions. Therefore, the scale used for the evaluation of adolescent asthma self-efficacy lacks the ability to predict any emotional function (Rhee et al., 2020). The ASE is mainly aimed at children aged 8–17 years, and not adolescents. We know explicitly that the experiences of adolescents are very different from those of young children (Orrell-Valente et al., 2008). In addition, the ASE failed to introduce a project determination method and the scale also lacked reliability data for retesting (Bursch et al., 1999). Therefore, a tool with more complete measurement content, being suitable to cater to different age groups, and designed in accordance with the development process of the program is needed to meet the evaluation needs of adolescent asthma self-efficacy programs.

Recently, in order to measure the self-management self-efficacy of asthmatic adolescents better, British scholars have developed the Adolescent Asthma Self efficacy Questionnaire (AASEQ) (Holley et al., 2019). The AASEQ is a disease-specific tool drawn up with 27 projects. Its development follows the guidelines for the development of contemporary scales, adopts the methods of literature review and qualitative research, and integrates the opinions of asthmatic adolescents, their parents, and relevant medical professionals. Although it has been proved to have satisfactory psychometric properties (Holley et al., 2019), the use of AASEQ in China is limited because it is a tool that has been developed and verified in Western culture. As far as we know, there is no verified version in Mandarin Chinese.

The purpose of this study was to first translate the AASEQ into Chinese under the authorization of the original researcher and then to evaluate the reliability and effectiveness of the Chinese version of the AASEQ.

## Methods

### Ethical considerations

This study was approved by the Research Ethics Committee of the Children's Hospital of Nanjing Medical University, Nanjing, Jiangsu Province, China. Before participation, the background and purpose of the present study were explained to asthmatic adolescents and their caregivers in detail. This study is voluntary and can be withdrawn at any time. The anonymity method is used to ensure the confidentiality of all participants of the study. The informed consent form was signed by all participants of the study. The data collected from the participants' replies are for the purpose of this study only.

### Design, participants, and data collection

A cross-sectional study was conducted at the outpatient asthma clinic located at the Children's Medical Center of Jiangsu Province from July 2021 to June 2022. The subjects were adolescents aged 12–18 years. All participants, i.e., adolescents, must have been diagnosed with asthma according to clear definitions or internationally recognized standards and should have no other chronic diseases that could have a significant impact on daily life.

Questionnaires including personal information forms, and the Chinese version of the AASEQ, were provided to adolescents and informed consent was provided to caregivers.

The sample size estimation was guided by the rule of thumb, and it required at least 10 respondents for each item in the confirmatory factor analysis (Tabachnick and Fidell, 2007). Taking the 27 items in AASEQ, the minimum sample size required is 270 participants. In order to obtain meaningful parameter estimates, at least 200 participants were required to perform the confirmatory factor analysis (Marsh et al., 1998). A total of 489 adolescents were invited to participate, and out of them 468 adolescents completed all the surveys, indicating that there are enough samples for exploratory factor analysis (EFA) and confirmatory factor analysis (CFA) (Polit and Yang, 2016). The reason why some patients refused to participate was the lack of time and interest.

### Instruments

#### Demographics and asthma information

Sociodemographic information of the participants, such as age, gender, educational background, and asthma-related information, including length of time since diagnosis, medication, severity, asthma frequency, and number of outpatient visits due to asthma within the past year, was collected by a purpose-built questionnaire.

#### Chinese general self-efficacy scale

The GSES was developed by Schwarzer (Schwarzer and Jerusalem, 1995) and consisted of 10 items. The scale uses a 4-point Likert-type scale ranging from 1 (never) to 5 (always). It has been validated in Turkey (Erci, 2006), Chile (Clavijo et al., 2020), and other countries. The Chinese translated version of the GSES was developed by Zhang and the Cronbach's α was 0.87 and the test-retest coefficient was 0.83 (Zhang and Schwarzer, 1995). They were both used for assessing the criterion validity of the translated version of the AASEQ in this study.

#### Adolescent Asthma Self-Efficacy Questionnaire

The AASEQ developed by Holley et al. (2019) is a self-reporting scale that includes medication, symptom management, asthma beliefs, friends-family, and school as key dimensions. It consists of 27 projects, each of which has a score of 0–100. The participants were asked to rate their confidence in completing each task, with 0 being completely incapable of doing it, 50 being generally able to do it, and 100 being very certain to be able to do it. All AASEQ answers are added together and then divided by 27 to get the overall average score (0–100). The sum of the subscale items is divided by the number of items in each subscale. Higher scores indicate greater self-efficacy in asthma management. In the UK, the Cronbach's alpha for the total scale was 0.91 and the test-retest coefficient was 0.82, indicating good psychometric properties.

### Translation and cultural adaptation of the instrument

We obtained authorization to use the original version of the AASEQ after contacting the author by email. Then, we followed the guidelines recommended by the American Academy of Orthopedic Surgeons (AAOS) for cross-cultural adaptation (Beaton et al., 2000). The cross-cultural adaptation process of the scale is “a process designed to maximize the attainment of semantic, idiomatic, experiential, and conceptual equivalence between the source and target questionnaires.” It consists of five steps: forward translation, synthesis of the translation, back translation, expert committee review, and test of the pre-final version (Beaton et al., 2000).

First, the scale was translated into simplified Chinese by two bilingual translators. One of the researchers was a graduate student of nursing from England, and the other was an English teacher. Next, the third translator compared the two versions and then compared them with the original scale to find out the differences in words, phrases, or meanings. Then, an eight-member committee, including the two translators and six authors of the study, analyzed and discussed any ambiguity issues that had arisen in translation, and even prepared the first draft of the instrument in Chinese.

After that, the other two translators who had never seen the original scale translated the integrated version into English independently. One of them was the deputy chief physician of the Department of Respiratory Medicine, a Ph.D., and the other was a masters graduate who lived abroad. After evaluating the semantic equivalence between the original scale and the two translated versions, the research team modified few words to make them more akin to and consistent with Chinese culture and also easier for the adolescents to fill in.

We designed an expert consultation letter to request experts to evaluate each item in the first version of the ASSEQ scale. Any suggestions from these experts were welcome. A total of seven experts were invited, which included clinical nursing experts, nursing educators, psychologists, and specialist nurse of respiratory medicine. They were required to evaluate the relevance and clarity of each item on a 4-point Likert scale (1 being irrelevant/clear to 4 being highly relevant/clear). For items rated 1 or 2, the experts were asked to come up with an alternative expression. Some items have been revised based on expert opinions and the cultural background of our country China. “I can control my asthma every day” and “I know when I am out of breath because of my asthma rather than because I feel a bit panicky” were revised to “I can control my asthma day-to-day” and “I know when I am out of breath because of my asthma rather than because I feel panicky.” The content validity of the questionnaire was evaluated by employing the item-level content validity index (I-CVI; reference range ≥ 0.78) and the scale average content validity index (S-CVI/Ave; reference range ≥ 0.90) (Polit et al., 2007).

A cognitive interview was conducted with 12 adolescents with asthma to identify how respondents understand the meaning of the questionnaire items and how to choose answers based on the understanding of the items, so as to reduce the errors that respondents may make when answering questions during the formal survey. The main questions in our interview included “Talk about your overall understanding and feelings of this sentence,” “What does this term mean to you?” “What can you recall when referring to this sentence?” “How did you get this answer?” and “Is it easy or difficult to choose this answer?” These adolescents were not included in the study sample. No revision was made according to the feedback, but only a preliminary version of 27-item C-AASEQ scales was formulated and sent to 10 healthcare providers (including nurses and doctors) for face validity evaluation.

### Data analysis

Statistical analysis was conducted using IBM SPSS STATISTICS 25.0 and AMOS 24.0. Descriptive statistics was used to assess the participants' demographic characteristics. Discrimination ability of the C-AASEQ scale was assessed by employing the item-total scale correlation, and the correlation coefficient below 0.3 was suitable for deleting items. Internal consistency reliability was measured using the Cronbach's α coefficients, and α values >0.8 were considered ideal (Terwee et al., 2007).

The EFA and CFA were used to examine the construct validity. The first part (*N* = 234) was used for exploratory factor analysis to explore the factor structure of C-AASEQ, and the second part (*N* = 234) was used for confirmatory factor analysis to confirm the results of EFA. Prior to EFA, Kaiser–Meyer–Olkin (KMO) and Bartlett sphericity tests were used to check the adequacy of sampling. Factors with a factor load >0.40 and an eigenvalue >1.0 were extracted. To test the goodness of fit, this study used chi-square/degrees of freedom (χ^2^/df, cut-off<3), root mean square error of approximation (RMSEA, cut-off<0.08), comparative fit index (CFI, cut-off≥0.95), goodness-of-fit index (GFI, cut-off≥0.85), and incremental fit index (IFI, cut-off≥0.90).

## Results

A total of 468 adolescents completed the survey, and the characteristics of the participants are shown in Table 1.

**Table 1:** Demographics of adolescents with asthma and family carers (*N* = 468).

	**Mean (SD) or *N* (%)**
**Adolescents with asthma**	
*N* = 468	
Age (years)	14.46 ± 1.83
Sex	
Female	191 (40.8)
Male	277 (59.2)
Family history	
Yes	161 (34.4)
No	307 (65.6)
Education	
Junior high school	242 (51.7)
Senior high school	218 (46.6)
University	8 (1.7)
Length of time since diagnosis years/year	9.13 ± 2.92
Total number of hospital visits due to asthma	7.59 ± 3.10
Severity	
Mild	409 (87.4)
Severe	59 (12.6)
Asthma attack frequency	2.69 ± 1.72
**Carergivers**	
Parents	348 (74.4)
Grandparents	104 (22.2)
Others	16 (3.4)
Family type	
Stem family	267 (57.1)
Core-family	189 (40.1)
Single-parent family	12 (2.8)

### Content and face validity

Item-level content validity index (I-CVI) was calculated as the number of experts giving a rating of either 3 or 4 divided by the total number of experts for each item. S-CVI was calculated by taking an average of the I-CVIs. Calculation results showed that I-CVI values of the C-AASEQ ranged from 0.86 to 1, and the S-CVI was 0.96, indicating acceptable content validity. Comments were also sought from healthcare providers on the clarity of the content. They generally stated that the questions of the C-AASEQ for adolescents were easy to understand and answer.

### Construct validity

Item-total correlation coefficients of the C-AASEQ scale ranged from 0.322 to 0.614, which were all statistically significant. If any item was deleted, the alpha value of the whole scale was decreased, indicating that all items were suitable for being included in the EFA. The KMO coefficient was 0.827, and Bartlett's sphericity test was significant (*p* < 0.001), which supported the effort taken for conducting an EFA.

The exploratory factor analysis suggested a four-factor solution, which explained 62.697% of the total variance. The factor loads of the four-factor model of the C-AASEQ ranged between 0.631 and 0.880. Table 2 details which items loaded on the four-factor.

**Table 2:** Item-total correlation, reliability coefficients, and factor loads of the Chinese Adolescent Asthma Self-Efficacy Questionnaire (C-AASEQ; *N* = 234).

**Factor name**	**Item**	**Item-total correlation**	**Factor loads**	**Cronbach's alpha if item deleted**	**Cronbach's alpha**
Medication	I know how to correctly use my asthma inhaler/spacer/medication	0.353^**^	0.784	0.861	0.810
	I know when to use my asthma medication	0.470^**^	0.817	0.858	
	I know which of my inhalers I need to take	0.322^**^	0.631	0.863	
	I know what my preventer inhaler is for	0.389^**^	0.785	0.861	
	I know what my reliever inhaler is for	0.425^**^	0.695	0.860	
Symptom management	I can be prepared to deal with an asthma attack	0.458^**^	0.789	0.859	0.905
	I know how to stay calm when I am having trouble breathing	0.514^**^	0.729	0.857	
	I know when I am out of breath because of my asthma rather than because of exercise	0.509^**^	0.798	0.857	
	I know when I am out of breath because of my asthma rather than because I feel panicky	0.426^**^	0.727	0.860	
	I know how to control my asthma when I am having trouble breathing	0.554^**^	0.819	0.856	
	I know when to use my inhaler to manage a serious breathing problem	0.553^**^	0.772	0.856	
	I know when I might need to go to hospital because of a serious breathing problem	0.562^**^	0.769	0.856	
	I know what to do to avoid triggers for my asthma	0.440^**^	0.756	0.860	
Asthma beliefs	I am in control of my asthma	0.363^**^	0.798	0.861	0.859
	I can do physical activity such as sports	0.329^**^	0.852	0.862	
	I can have a normal life	0.377^**^	0.800	0.861	
	I can do the things that I want to do	0.340^**^	0.714	0.862	
	I can control my asthma day-to-day	0.398^**^	0.785	0.860	
Friends, family and school	I can take my inhalers in front of my friends	0.479^**^	0.801	0.858	0.927
	I can take my inhalers around other people at school	0.576^**^	0.771	0.855	
	I can talk honestly to my friends about my asthma	0.506^**^	0.829	0.857	
	I can talk honestly to my parents about my asthma	0.552^**^	0.844	0.856	
	I can talk honestly to my doctor or nurse about my asthma	0.528^**^	0.771	0.857	
	I can talk honestly to my teachers about my asthma	0.506^**^	0.744	0.857	
	I can ask my parents for help if I am having trouble breathing or having an asthma attack	0.523^**^	0.811	0.857	
	I can ask my teachers for help if I am having trouble breathing or having an asthma attack	0.614^**^	0.706	0.854	
	I can ask my friends for help if I am having trouble breathing or having an asthma attack	0.515^**^	0.880	0.857	
Total					0.863

** *p*, level of statistical significance; significant (*p* < 0.01).

**Figure 1:**
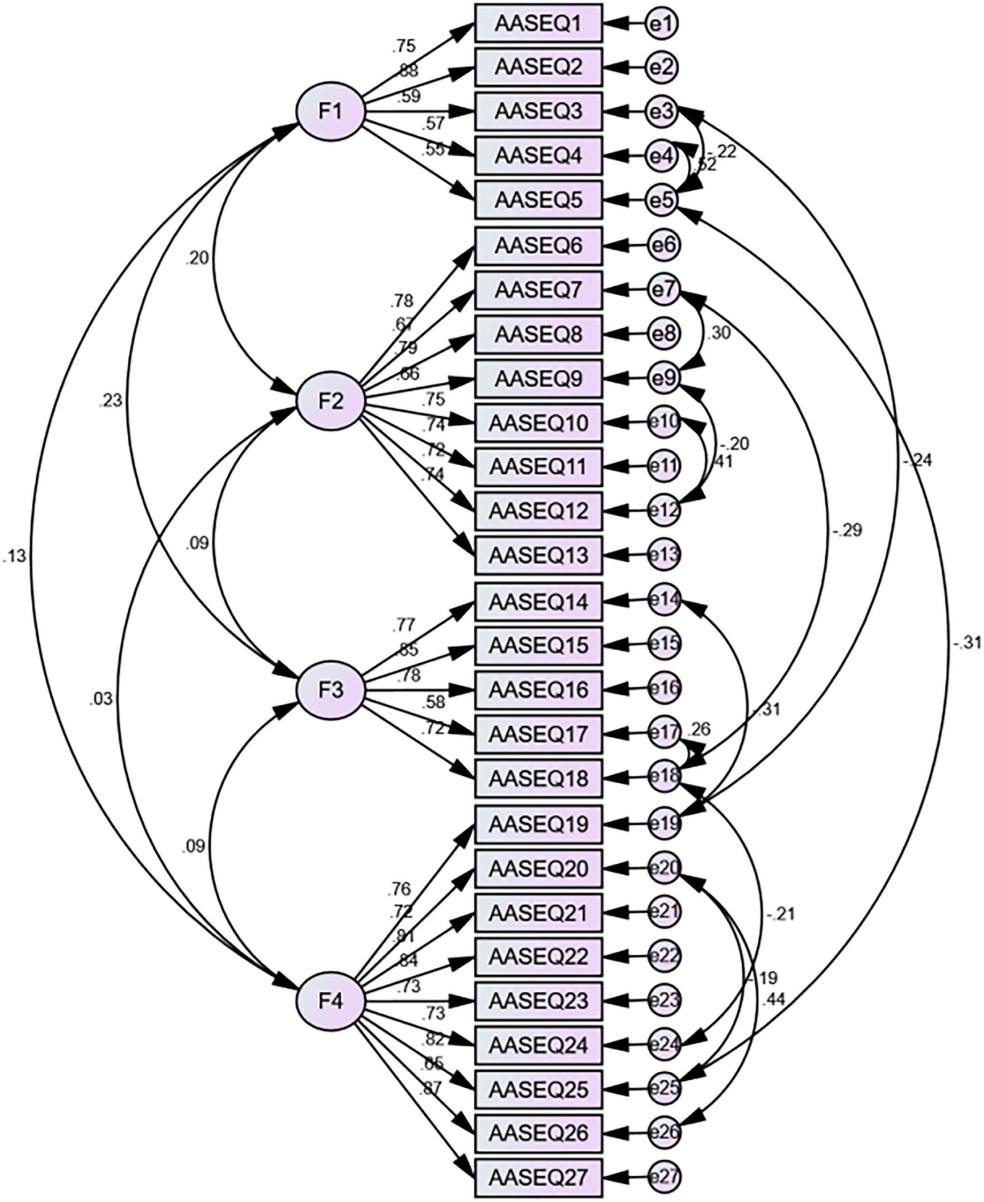
The structure of AASEQ.

Part 2 data were used to examine the four-factor model by the CFA (*N* = 234). Results of the CFA showed that the primary model had a poor data fit, with results including χ^2^/df = 2.755, RMSEA = 0.087, CFI = 0.847, GFI = 0.847, IFI = 0.848, and TLI = 0.831. Thus, we calculated the modification index (MI) between each of the two items in all the items in AMOS. And then, we established error covariances between the following items in AMOS according to the modification indices (the process is shown in Figure 1): e3 and e5, e3 and e19, e4 and e5, e5 and e25, e7 and e9, e7 and e18, e9 and e12, e10 and e12, e14 and e19, e17 and e18, e18 and e24, e20 and e25, and e20 and e26, respectively. Based on the goodness-of-fit statistics, the secondary model had acceptable fit (Figure 1), with results including χ^2^/df = 1.887, RMSEA = 0.062, CFI = 0.926, GFI = 0.856, AGFI = 0.86, IFI = 0.927, and TLI = 0.915. Table 3 shows the model fit indices of the C-AASEQ for the primary and secondary models.

**Table 3:** Fit indices of the models (*N* = 234).

**Fit indices**	**Good fit**	**Acceptable fit**	**four-factor model**	**Adjusted four-factor model**
χ^2^/*df*	1 < χ^2^/*df* < 3	3 < χ^2^/*df* < 5	2.755	1.887
RMSEA	0 ≤ RMSEA ≤ 0.05	0.05 < RMSEA < 0.08	0.087	0.062
CFI	0.97 ≤ CFI ≤ 1	CFI ≥ 0.90	0.847	0.926
GFI	0.90 ≤ GFI ≤ 1	0.85 ≤ GFI < 0.90	0.792	0.856
IFI	0.95 ≤ IFI ≤ 1	IFI ≥ 0.90	0.848	0.927
TLI	0.95 ≤ TLI ≤ 1	TLI ≥ 0.90	0.831	0.915

χ^2^/df, chi-square/degree of freedom; RMSEA, root mean square error of approximation; CFI, comparative fit index; GFI, goodness-of-fit index; IFI, incremental fit index, TLI, Tucker–Lewis index.

### Cross-sectional validity of C-AASEQ

With the GSES as the calibration tool, Pearson's correlation analysis showed that the total score of the Chinese version of AASEQ was positively correlated with the total score of the GSES (*r* = 0.590, *p* < 0.01). The scores of the four subscales were also positively correlated with that of GSES, and the *r* ranging from 0.315 to 0.448 (*p* < 0.01; Table 4).

**Table 4:** Pearson's correlations between the Chinese Adolescent Asthma Self-Efficacy Questionnaire (C-AASEQ) scale and subscales and the Generalized Self-Efficacy Scale (GSES).

	**C-AASEQ total**	**C-AASEQ subscales**			
		**Medication**	**Symptom management**	**Asthma beliefs**	**Friends, family and school**
GSES	0.590^**^	0.448^**^	0.385^**^	0.315^**^	0.345^**^

Data are presented as Pearson's correlation coefficients to assess how well the C-AASEQ agrees with GSES.

** *p* < 0.01.

### Consistency over time of the C-AASEQ

We took a 2-week test–retest reliability of the C-AASEQ in 122 adolescents. The ICC was 0.90 for the total scale and 0.64 to 0.89 for the four subscales. All the values were above 0.60, indicating stability of the measure.

## Discussion

Recent studies have emphasized the importance of self-efficacy in disease self-management of asthmatic adolescents (Sleath et al., 2022). Therefore, it is crucial to choose scientific and effective evaluation tools to evaluate adolescents' self-efficacy for providing cost-effective clinical nursing interventions. The AASEQ scale was translated into simplified Chinese in our study and 468 adolescents with asthma were invited to test the psychometric properties of the C-AASEQ scale. As the participants reported, the scale items were easy to understand, which indicated that C-AASEQ is a feasible and appropriate tool to measure the self-efficacy of adolescents with asthma.

Overall, the results of this study showed that the C-AASEQ for asthmatic adolescents had a convincing reliability and validity in the sample recruited in this study. All the item-total correlation coefficients were above 0.3, which means a good discriminating ability. Besides, the Cronbach's α coefficient of the total scale was 0.863, slightly lower than that of the original version, which was 0.91 (Holley et al., 2019). The differences in research results may have been caused by different cultures and activities in different countries. In our study, the included participants were recruited face-to-face in the asthma-specific clinic of a tertiary children's hospital in China. All of them had a clear diagnosis of asthma and were recruited before or after the visit. They were accompanied by their parents or other adults, and the study had a high response. In the original version, 14% of the participants were recruited through social media (Holley et al., 2019), who, on the one hand, may not have asthma, and, on the other, caused a larger selection bias, because more active adolescents were more actively involved in the study, they tended to have better asthma self-management. The source of different subjects and their different enthusiasms for participation may have an impact on the psychological measurement of the scale. In addition, the healthcare systems in China differ from those in source countries, and there are significant differences in the medical resource support that adolescents receive, which can also have an impact on adolescent asthma self-management.

The Cronbach's alpha values of the four-factor model were between 0.810 and 0.927, indicating that there was an acceptable internal consistency reliability of the C-AASEQ among Chinese adolescents with asthma.

Twenty-seven items were grouped under four factors by EFA results, explaining 62.697% of the total variance. The factor loads are considered ideal for all the items which were 0.40 or higher (Field, 2013). The fit of the initial model was less ideal due to which some residuals were correlated. Therefore, the primary model was adjusted based on modification indices, and error covariances were established between some items.

Retest reliability means using the same scale at two different times to survey the same group of respondents to measure their stability over time. Two weeks was chosen as the retest time point, on the one hand, to avoid the influence of environmental changes on the measurement results which could be caused due to too long time; on the other hand, to avoid the influence of too short time on the measurement results caused by adolescents remembering the previous answers. There was a strong ICC of 0.90 between the baseline total score and the retest total score, indicating that there was an excellent test-retest reliability of the C-AASEQ.

The C-AASEQ for asthmatic adolescents has been proved to have positive reliability and validity, and can be used to evaluate the self-management self-efficacy of asthmatic adolescents. Therefore, future research can use C-AASEQ to provide support for the construct of self-efficacy of adolescents with asthma.

We recommend that adolescents complete the questionnaire while waiting for outpatient consultation. The results can reflect the weaknesses of disease self-management of adolescents and the areas where they need guidance and support the most. The findings can be used by clinicians for targeted health education, which can ensure that the needs of adolescents are not ignored. We believe this tool can also be used by researchers and educators working with asthmatic adolescents to identify areas where adolescents lack confidence in asthma self-management, so as to guide them in specific asthma management education and strategies. Although the questionnaire can be completed within 5–15 min, in practice, too many scale questions may affect the response quality of the scale, so a shorter version of the scale should be considered. The scale is a self-report tool for adolescent asthma self-management self-efficacy, which should be combined with objective evaluation indices and other evaluation indices to ensure the scientific and comprehensive evaluation results.

This study still has certain limitations. We recruited adolescents with asthma from only one medical center in China, the use of a convenience sample, which may have affected its generalizability. Moreover, we did not investigate factors influencing self-efficacy among adolescents with asthma, which will be important for our future studies. In addition, as this study was a cross-sectional survey, it has not been clearly revealed as to how the self-efficacy of asthmatic adolescents changes over time.

## Data availability statement

The raw data supporting the conclusions of this article will be made available by the authors, without undue reservation.

## Ethics statement

Written informed consent was obtained from the minor(s)' legal guardian/next of kin for the publication of any potentially identifiable images or data included in this article.

## Author contributions

YW and HC are responsible for the conception and design of the research and drafted the article. YW, JW, and JC contributed to data collection. HC and RJ contributed to the analysis and interpretation of the data. JC and ML reviewed and critically revised the content of the study. ML finally approved the version to be published. All authors contributed to the article and approved the submitted version.
